# Vitamin D and Skin Cancer: An Epidemiological, Patient-Centered Update and Review

**DOI:** 10.3390/nu13124292

**Published:** 2021-11-28

**Authors:** Alejandro Martin-Gorgojo, Yolanda Gilaberte, Eduardo Nagore

**Affiliations:** 1 STI/Dermatology Department, Madrid City Council, 28006 Madrid, Spain; 2 Dermatology Department, Hospital Universitario Miguel Servet, IIS Aragon, 50009 Zaragoza, Spain; ygilaberte@gmail.com; 3 Dermatology Department, Universidad Catolica de Valencia, 46001 Valencia, Spain; eduardo.nagore@ucv.es

**Keywords:** vitamin D, vitamin D deficiency, skin neoplasms, melanoma, cutaneous malignant melanoma, basal cell carcinoma, squamous cell carcinoma, ultraviolet rays, primary prevention, prevention and control

## Abstract

Background: The current vitamin D deficiency epidemic is accompanied by an increase in endemic skin cancer. There are still multiple controversies. This review aims to give practical recommendations regarding vitamin D among people at risk or with a personal history of skin cancer. Methods: Narrative review including human research articles published between 2011 and 2021, elaborated bearing in mind an epidemiological, patient-centered approach. Results: Ultraviolet (UV) exposure (neither artificial nor natural) is not the ideal source to synthesize vitamin D. There is conflicting epidemiological evidence regarding vitamin D, non-melanoma skin cancer (NMSC), and cutaneous melanoma (CMM), confounded by the effect of sun exposure and other factors. Conclusions: Current evidence is controversial, and there are no widely applicable strategies. We propose three practical recommendations. Firstly, sun protection recommendations should be kept among people at risk or with a personal history of skin cancer. Secondly, vitamin D should preferably be sourced through diet. In patients with melanoma or at risk of cutaneous cancer, serum vitamin D checks are warranted to detect and avoid its insufficiency.

## 1. Introduction

Misnamed vitamin D is a true hormone that humans can synthesize upon sun exposure or through a balanced and healthy diet including vitamin D-rich foods or supplements. However, our current predominantly indoor lifestyle with unhealthy, intense, and sporadic sun exposure, along with other factors (such as human migratory movements), have contributed to the vitamin D deficiency epidemic. Paradoxically, this epidemic is accompanied by an increase in endemic skin cancer [1].

Vitamin D shows anti-proliferative and pro-apoptotic effects in vitro on both melanocytes and keratinocytes. Ultraviolet (UV) exposure is the leading environmental risk factor for cutaneous malignant melanoma (CMM) and non-melanoma skin cancer (NMSC). Some studies have observed that vitamin D synthesis may protect against NMSC. However, the optimum vitamin D dose to reduce skin cancer risk has yet to be confirmed [2].

There are still multiple controversies regarding vitamin D and skin cancer, including the recommended serum levels and the limiting role that preventive measures against skin cancer may have on effective vitamin D synthesis.

This review aims to address practical recommendations regarding:

- The effect on vitamin D of sun protection recommendations among people at risk of skin cancer or patients with a personal history of skin cancer;
- The best source to acquire adequate vitamin D levels;
- The current evidence regarding vitamin D, non-melanoma skin cancer, and cutaneous melanoma.

## 2. Methods

A narrative review was performed, including PubMed-indexed human research articles published between 2011 and 2021, with available abstracts and written in English, Spanish, or French. The following search query was used: “(“Vitamin D” [Mesh] OR “Vitamin D Deficiency” [Mesh] OR Cholecalciferol [Mesh]) AND (“Skin Neoplasms” [Mesh] OR “Melanoma” [Mesh] OR “Carcinoma, Basal Cell” [Mesh] OR “Carcinoma, Squamous Cell” [Mesh]) AND (“prevention and control” [Subheading]).”

The review was elaborated bearing in mind an epidemiological, patient-centered approach. From a total of 230 initially screened publications, we selected those that specifically addressed the topics covered by this review. We read the full text of 102 articles, extracted some other references from them, and finally, included 62 references (included in this number is an additional one suggested during peer-review of the manuscript).

## 3. “Sun-Related” Cancer: Magnitude of the Problem

Basal cell carcinoma (BCC), squamous cell carcinoma (SCC) —these first two are usually classified as NMSC–, and cutaneous malignant melanoma (CMM) are the three most frequent types of cutaneous cancer, which are considered “sun-related” (mainly UV-related) cancers.

A significant proportion of national tumor registries do not consider NMSC, given their high frequency and apparently low impact, as well as the fact that some cases are treated without histological confirmation. However, NMSC constitute the most commonly diagnosed cancers in North America, Australia, and New Zealand [3].

An estimated number of 1,042,056 new NMSC cases were diagnosed worldwide in 2018, with 65,155 deaths (approximately 6%) attributable to NMSC (mostly SCC) [3]. The incidence of SCC seems to be increasing, whereas mortality remains stable [4].

Cutaneous melanoma is considered the most lethal of the three main types of skin cancer. An estimated 287,723 cases of CMM were diagnosed worldwide in 2018, causing up to 60,712 deaths (21% mortality) [3]. CMM incidence has increased in most developed countries, mainly accounting for thinner lesions with better prognoses. Mortality tends to be stable among females in the United States (1.9 deaths per 100,000 attributed to CMM) but has been increasing over the years among males (from 2.88 per 100,000 in 1975 to 4.44 per 100,000 in the 2011–2015 period). These differences among male and female rates are controversial; behavioral, genetic, and hormonal factors may play a role [4].

## 4. The Vitamin D Deficiency Epidemic: The Problematic Effects of UV Exposure, UV Protection, and Sun Avoidance

Vitamin D is a prohormone with two major forms: D_3_ (cholecalciferol) and D_2_ (ergocalciferol). Vitamin D_2_ or D_3_ can be acquired exogenously from dietary intake. Vitamin D_3_ may also be synthesized endogenously by photochemical modification of 7-dehydrocholesterol in the skin upon UVB radiation. This cutaneous synthesis depends on different factors: the solar UV index, the amount of sun-exposed and sun-protected skin, the amount of time under the sun, the body mass index, the age, and skin phototype [5].

Vitamin D deficiency is an increasingly concerning global issue. Optimal levels are not well defined, even for bone metabolism, and especially among non-Caucasians, who usually have greater bone mass despite generally having lower serum vitamin D levels. Among Caucasians, vitamin D levels tend to be lower in the fairest phototypes. This is partially linked to less sun exposure given their higher photosensitivity, but there are other factors involving vitamin D metabolism [6]. However, certain populations show higher serum vitamin D among individuals with the fairest phototypes and pigmentation traits: a UK-based cohort observed this, agreeing with the vitamin D hypothesis for the evolution of skin pigmentation [7].

UVB radiation (280–320 nm) is considered the most important environmental risk factor for all skin cancers. UV wavelengths longer than 320 nm (UVA) are linked to potentially mutagenic oxidative DNA damage. UV wavelengths shorter than 280 nm (UVC) are blocked by the ozone layer [8].

UV rays induce multiple molecular cellular signaling events, resulting in inflammation and secondary immunosuppression (with failure of apoptosis and aberrant differentiation) [2]. UV-induced DNA damage induces the formation of cyclobutane pyrimidine dimers (CPD) and pyrimidine(6–4)pyrimidone photoproducts. These can be highly mutagenic if they are not repaired before cell division, causing DNA damage (which is said to have the “UV signature”), leading to UV-induced immunosuppression and potential carcinogenesis [9]. These mutations are frequently identifiable in oncogene and tumor suppressor genes (such as p53) in skin tumors such as BCC, SCC, and actinic keratoses [8]. They are also frequently identified in the telomerase reverse transcriptase (TERT) promoter gene, both in NMSC and CMM [10,11,12].

Cumulative UV exposure results in mutagenesis and, secondarily, cancer through a complex interaction with other factors [2].

### 4.1. Type of Sun Exposure and Implications

There are two main categories of sun exposure:

(a) Intermittent sun exposure. Currently the most frequent type of sun exposure. Typical of indoor workers who go outdoors on the weekend, sunbathe or have vacations in sunny places;
(b) Chronic sun exposure, which is usually associated with occupational exposure.

Sunburns (and intermittent sun exposure) increase the risk of CMM, mainly of the superficial-spreading subtype, especially if these are severe and occur before 18 years of age. Chronic sun exposure may lead to photoaging, cutaneous immune suppression, NMSC, and lentigo maligna CMM subtype.

The timing of the sun exposure is also relevant. It appears that sun exposure during the early years of life plays a crucial role in increasing the risk of melanoma in adulthood. Cell growth during youth may multiply the risk. However, according to some authors, high levels of early life sun exposure correlate to high levels of sun exposure over the lifetime [4].

Furthermore, our lifestyle changes, including frequent use of multiple electronic devices, have extended our artificial light exposure and may have consequences in the circadian control of the skin [13].

### 4.2. Vitamin D and the Need for Sun Exposure?

Around 90% of vitamin D is produced in the skin upon exposure to sunlight, mainly to the ultraviolet type B (UVB) spectrum. A meta-analysis including 14 studies from northern Europe and one from New Zealand showed that partial skin exposure (of less than 10% of the skin surface) to single UVB doses of 0.75 to 3 standard erythematous doses (SED) are effective to generate or maintain a healthy vitamin status [14]. Nevertheless, excessive solar exposure, particularly to this part of the UV spectrum, can be problematic.

Vitamin D deficiency in western developed countries occurs more frequently among individuals with darker phototypes, but also in most of the active adult population (indoor workers with scarce casual UV exposure) and elder people (with decreased skin thickness and less skin vitamin D synthesis capacity) [15]. A study observed that most North American children are not receiving enough UVB throughout the year to meet their minimal vitamin D requirements [16]. However, among children of a significant proportion of populations, sun exposure cannot be considered a good source of vitamin D. A Polish study including a cohort of 32 children who had attended a summer camp on the Baltic Sea, with low daily sun exposure, observed that their serum vitamin D_3_ levels improved modestly (24%), in a proportion that did not reach that of the increase in urinary CPD secondary to sun damage (1262%) [17]. This was equivalent to the level of CPD of adults who received higher UV doses during a shorter holiday in Tenerife [18]. 

It is, therefore, preferable and safer to obtain adequate levels of vitamin D through diet than through sun exposure. In fact, it is currently accepted that dietary and supplemental vitamin D is functionally identical to that produced after UV exposure, being more reliable and quantifiable (the risks of keeping high levels of vitamin D have not been extensively studied) source of this vitamin [19].

If sun exposure continues to be prescribed, it is paramount to find a healthy balance that maximizes vitamin D synthesis while minimizing skin cancer risk [1,20]. Correct medical advice can be of help to achieve it. Given that the UVB:UVA ratio is maximal at noon, some authors have proposed that the best way to obtain vitamin D through sun exposure with minimal carcinogenic risk is by getting sun-exposed in the middle of the day (avoiding sunburn), rather than in the afternoon or morning [21]. 

There seems to be little to no consensus among different specialists (namely dermatologists, endocrinologists, and family medicine doctors) regarding the amount of sun exposure time they recommend their patients to synthesize enough vitamin D [22]. According to a 500 Australian general practitioners survey, non-dermatologist physicians tend to be more concerned about vitamin D deficiency than skin cancer, which may cause to advise too much sun [23].

Population-wide campaigns can also be helpful. An Israel-based study observed reasonable knowledge on the beneficial and deleterious effects of sun exposure among the general population [24]. The latter seems to be an exception since other studies have identified that men in lower socio-economic groups have worse awareness and behavior regarding sunlight exposure and vitamin D [25].

In any case, advice on sun exposure should be adjusted to each patient’s characteristics, along with the specific geographic and climatic conditions. Understanding the UV index and the importance of sun exposure duration should be incorporated in our advice [26]. Computerized decision aids and algorithms that take into account these complexities may be useful [23]. There are also some promising experiences using wearable devices to promote UV exposure awareness [27]. Finally, it is necessary that political institutions and health societies, along with institutions, find common ground and common language to inform the general public without confusing them [28]. We need this to be more effective and adaptable than ever, given that climate change and stratospheric ozone modification have health effects and change UV exposure patterns [29].

### 4.3. Tanning Sunbeds: An Unhelpful Resource

Sunbeds deliver as much as 99% UVA (in excessive dosages, which are calculated to be 5 to 15 times greater than the amount of summer midday sun on a Mediterranean beach), but also emit small quantities of UVB, which are necessary to induce long-lasting tan, and may also increase serum vitamin D [5]. This has led to propose the use of sunlamps to achieve better vitamin D levels. There is proof that certain health professionals, such as nurses, have used them personally, with this effect [30]. The problem with sunbeds is that they only increase vitamin D levels mildly and transiently. Therefore, these devices are not a safe source and should not be considered an option to achieve them [31].

Indoor tanning has proven to increase the risk of BCC, SCC, and premature photoaging. This risk is higher for squamous cell carcinoma than for basal cell carcinoma [32]. Regarding CMM, the results of a meta-analysis including 28 case-control studies found a mild increase in melanoma risk considering sunbed use anytime in life, with more significant figures when considering sunbed use before 35 years of age (relative risk of 1.59). This study found a dose-response relation between the amount of sunbed use and the risk of melanoma and an estimated 3438 new sunbed-associated melanoma cases in Europe [33]. However, there are confounding factors such as the fact that “light-seeking” behaviors are strikingly different among countries according to the latitudes, causing people with a higher risk of skin cancer (with an inability to tan, freckles, and red or blond hair) to be frequent users of sunbeds [6]. Furthermore, indoor tanners are more likely to be outdoor tanners [34].

Taking into account these data, recreational indoor tanning can be considered a health hazard. The World Health Organization classified UV-emitting tanning devices as group 1 carcinogens. Many countries have banned sunbed use by children and are establishing regulations for sunbed providers [6,31].

Nevertheless, UV-emitting devices can be used to treat medical conditions such as psoriasis or atopic dermatitis. These patients may be treated with UVA (with or without oral photosensitizers such as psoralens) or narrow-band UVB phototherapy. The latter is safer in the long run since UVA with oral psoralen administration is shown to increase SCC risk among psoriasis patients (7) clearly and should be used with caution.

### 4.4. Sunscreens and Preventive Sun Avoidance as a Potential Part of the Problem?

Current sunscreens filter UVB and UVA with a better benefit/risk ratio than former UVB-blocking organic filters. They are useful to prevent sunburns. Although incomplete, there are enough data to affirm they are also helpful to prevent SCC, AK, and skin photoaging. However, to date, sunscreens have not proved their effectiveness in significantly preventing either CMM or BCC [15].

The limitation of perfect sunscreen use is that it may impair vitamin D synthesis [15] (with studies stating that sun protection factor (SPF) 15 application can reduce it up to 98% [35]), triggering some authors to express an urgent need to evaluate the long-term effect of recommending strict sun avoidance and extensive use of sunscreens in Caucasian populations [6].

However, some studies have observed that this vitamin D synthesis impairment by sunscreens is inconclusive and may not be significant in real-world conditions [35,36], though there have been few trials of the high sun protection factor sunscreens that are currently recommended widely [37].

A manuscript comparing adults in Kuwait who were regular sunscreen users with age, phototype, and sex-matched people who never used sunscreen showed no differences in vitamin D serum levels. The lack of significant differences in adults can be explained by the fact that sunscreen users tend to overexpose to the sun (given that UVB-sunburn appears much later or does not appear at all), in part counter-acting their beneficial effects [24]. Furthermore, the previously mentioned UK cohort of both parents and children showed that those with fairer skin that were regular sunscreen users maintained similar vitamin D levels as those with similar phototype and skin pigmentation traits that did not use sun protection [7].

People who have fairer skin and are more sun-sensitive should avoid sunburns by all means [38], as do patients at risk for skin cancer, such as solid organ transplant recipients [39]. In any case, it seems necessary to assess vitamin D status to maximize the benefits of sunscreen (32) and include information about vitamin D in skin cancer prevention information and campaigns [34,40].

## 5. Vitamin D Status and Supplementation in Carcinogenesis and Skin Cancer

In vitro and preclinical animal models have shown that vitamin D alters cancer cell differentiation, proliferation, and apoptosis, making it a candidate agent for cancer regulation [41]. Whether vitamin D prevents cancer in humans or limits cancer progression remains unresolved [42].

The role of vitamin D in cutaneous carcinogenesis is most likely related to its effects on the regulation of growth, cell death, angiogenesis, and cell differentiation. The vitamin D receptor (VDR) is codified by a gene located on chromosomal region 12q13, has variants that are thought to alter its function [1], and is increasingly being considered as a tumor suppressor in the skin (with protective actions against UV-induced epidermal cancer formation) [43].

The protective actions of vitamin D against cutaneous cancer have been evaluated from the study of the relationship between vitamin D (levels, polymorphisms in the vitamin D receptor, and dietary supplementation) with the incidence and survival of various neoplasms [1].

It has been repeatedly suggested that sun exposure, through vitamin D production, may yield a protective effect on various internal cancers. However, from a nested case-control study of Swedish population-based registries comparing more than 100,000 patients with basal cell carcinoma (as a paradigmatic example of people with more sun exposure and vitamin D produced through it) with about 1 million control patients, it could be found that patients with BCC are at higher risk of having other cancers before BCC diagnosis. This evidence contradicts that vitamin D production via regular sun exposure has protective effects on internal cancers [44].

Attempts have been made to determine the most appropriate cancer-protective vitamin D daily intake. Daily doses of 1500 international units (IU) of vitamin D_3_ were shown to reduce the male cancer mortality rate by 30% in the United States [1].

In recent years, many studies have made efforts to relate blood levels of vitamin D_3_ (25-OH vitamin D) to the incidence of some cancers. For these studies, minimum values of 30–35 ng/mL (75–87.5 nmol/l) were used as a reference, which are considered optimal for obtaining the maximum beneficial effects of vitamin D. These persist even after adjustment for factors that could influence vitamin D levels, such as body mass index or age.

Sufficient vitamin D serum levels confer protection against multiple malignancies. This is proved clinically in different tissues and in vitro in animal and cell culture studies. However, there is not enough epidemiologic evidence to support the positive role of vitamin D in preventing skin cancer, and there is even conflicting evidence [1,45]. As a matter of fact, a recent meta-analysis including 13 prospective studies suggested that vitamin D status is associated with greater risks of CMM and NMSC: each 30 nmol/L increment in 25(OH)-D_3_ levels was associated with a 42%, 30%, and 41% increase in the risks of CMM, SCC, and BCC. These increases were probably confounded by sun exposure [46].

### 5.1. Vitamin D and Non-Melanoma Skin Cancer

Higher serum vitamin D_3_ levels are associated with NMSC (OR: 2.07, CI: 1.52–2.80) [8,45], with a linear dose-response [46]. As previously stated, this is probably related to the dual effect of UVB, which allows vitamin D synthesis but, in turn, generates DNA damage causing skin cancer.

Even though xeroderma pigmentosum patients (with probably the highest risk of NMSC) have a high prevalence of vitamin D deficiency [47], current evidence regarding vitamin D and NMSC is controversial, and it is yet to be defined if vitamin D may decrease NMSC incidence or severity [2].

#### 5.1.1. Basal-Cell Carcinoma (BCC)

Vitamin D inhibits the hedgehog pathway (the key tumor pathway in the development of BCC). However, current epidemiological evidence is conflicting, and ad hoc, prospective studies in humans are needed to know the true relationship between vitamin D serum levels and BCC risk [48].

Apart from the linear dose-response increase in BCC risk regarding serum levels, the previously referenced meta-analysis showed a slightly higher risk of BCC among those receiving at least 100 daily international units of either dietary or supplemental vitamin D (RR: 1.02, CI: 1.00–1.03, *p* = 0.03) [46]. The secondary analysis of a randomized clinical trial of supplementation with vitamin D and/or calcium also showed no benefit in preventing BCC (HR: 0.99; 95% CI: 0.65–1.51) [49].

Conversely, a study observed that maintaining serum vitamin D_3_ levels above 25 ng/mL may significantly reduce recurrence rates of BCC [50].

#### 5.1.2. Squamous-Cell Carcinoma (SCC)

Molecular studies have shown that the VDR is induced by the tumor suppressor gene p63, which (along with p53) is critical for keratinocytes to initiate the DNA repair process after UV exposure [48].

Despite the observed increase in SCC incidence in those with higher vitamin D serum levels, probably confounded by excessive photodamage [46], there is starting to be some epidemiologic evidence to believe that vitamin D and/or calcium) supplementation may be useful to prevent SCC (HR: 0.42; 95% CI: 0.19–0.91) [49].

Additionally, some studies have assessed the usefulness of vitamin D supplementation or topical application in different indications. An example is the intermittent supplementation of cholecalciferol, which has proven to be helpful to enhance photodynamic therapy to treat squamous cell carcinoma [51].

### 5.2. Vitamin D and Cutaneous Melanoma

The vitamin D pathway may play a role in melanoma since VDR expression is detected in different melanoma samples and cells. Calcitriol is shown to inhibit tumor invasion and angiogenesis in melanoma cell lines in animal models [48].

Adequate vitamin D levels are associated with diminished risk of melanoma occurrence (RR 0.62 [0.42–0.94]) [45], although there are heterogeneous and conflicting results in different studies with various risk measures [46,48].

Regarding melanoma prognosis, lower serum vitamin D_3_ levels are significantly related to worse prognostic traits, namely Breslow thickness, along with poorer melanoma survival, even adjusting for inflammatory biomarkers [52]. Several studies have shown similar associations: one studied patients with variations in the gene coding for vitamin D-binding protein predisposing to lower serum vitamin D levels, with poorer melanoma-specific survival [53], and another confirming a significant association between vitamin D levels at diagnosis and location, tumor mitotic rate, and ulceration [54], and a more recent one observing vitamin D levels < 9.25 ng/mL as independent prognostic factor for overall survival in melanoma patients, linked to histologic ulceration [55]. Likewise, low vitamin D levels are related to increased susceptibility to melanoma, along with reduced melanoma survival [6]. However, several large-scale studies have not been able to prove the same associations [56].

Further investigation is warranted to determine whether supplementation of vitamin D could be of help for patients with or at risk of melanoma [52]. A study recently published in this journal confirmed the safety of vitamin D_3_ supplementation (100,000 international units every 50 days) to stage II melanoma patients. It also observed that Breslow thickness influences both disease-free survival and the response (in terms of serum vitamin D levels) to supplementation [57]. Lower melanoma incidence has been observed in patients following a vitamin D-rich diet, but it has not been confirmed in case-control studies including individuals with a diet rich in vitamin D and patients receiving supplements. This may be related with polymorphisms of the VDR receptor, which influence the antitumor role of vitamin D [58]. There is additional conflicting evidence: a study observed that high vitamin D intake resulted in an increased risk of melanoma among men but had a protective effect against invasive melanoma in women [59].

In any case, it is reasonable to give vitamin D supplementation to those with insufficient vitamin D levels and to perform regular serum vitamin D re-screening among patients with or at risk of melanoma [60,61].

## 6. Conclusions

Vitamin D is a hormone with proven in vitro anti-carcinogenic effects. Current evidence is controversial and there are no widely applicable strategies. According to the latest consensus statement from the second International Conference on Controversies in vitamin D, serum vitamin D levels < 50 nmol/L are likely to have adverse effects on health and affect one-quarter of the world’s population. This consensus also proposes ideal standards of further randomized controlled trials to evaluate the health benefits of vitamin D supplementation [62].

In the light of the reviewed manuscripts, we propose three practical recommendations:

- Sun protection recommendations among people at risk of skin cancer or patients with a personal history of skin cancer should be kept.
- Neither natural nor artificial sun exposure should be encouraged as the main source of vitamin D. Given that dietary and supplemental vitamin D is functionally identical to that produced after UV exposure (and is also more reliable and quantifiable), it should be the preferred source of this vitamin.
- In patients with melanoma, or at risk of cutaneous cancer, serum vitamin D checks are warranted in order to detect and avoid its insufficiency.

## Data Availability

A publicly available bibliographic database, PubMed.gov, was used in this study. The complete search query is specified in the Methods section of the article. The full bibliographic reference list is available on request from the corresponding author.
